# The Role of LncRNAs in Translation

**DOI:** 10.3390/ncrna7010016

**Published:** 2021-02-20

**Authors:** Didem Karakas, Bulent Ozpolat

**Affiliations:** 1 Department of Molecular Biology and Genetics, Faculty of Arts and Sciences, Istinye University, Istanbul 34010, Turkey; didemmkarakass@gmail.com; 2 Department of Experimental Therapeutics, The University of Texas MD Anderson Cancer Center, Houston, TX 77030, USA

**Keywords:** non-coding RNAs, long non-coding RNAs, ncRNAs, translation, cancer

## Abstract

Long non-coding RNAs (lncRNAs), a group of non-protein coding RNAs with lengths of more than 200 nucleotides, exert their effects by binding to DNA, mRNA, microRNA, and proteins and regulate gene expression at the transcriptional, post-transcriptional, translational, and post-translational levels. Depending on cellular location, lncRNAs are involved in a wide range of cellular functions, including chromatin modification, transcriptional activation, transcriptional interference, scaffolding and regulation of translational machinery. This review highlights recent studies on lncRNAs in the regulation of protein translation by modulating the translational factors (i.e, eIF4E, eIF4G, eIF4A, 4E-BP1, eEF5A) and signaling pathways involved in this process as wells as their potential roles as tumor suppressors or tumor promoters.

## 1. Introduction

The majority of the mammalian genome consists of non-coding RNAs (ncRNAs), including long ncRNAs (lncRNAs), transfer RNAs (tRNAs), ribosomal RNAs (rRNAs), and small ncRNAs such as microRNAs (miRNAs), small nuclear RNAs (snRNA) and circular RNAs (circRNAs), while only a small portion (~1.5%) of it is comprised of protein-coding mRNAs [1]. 

lncRNA transcripts, which are a group of ncRNAs longer than 200 nucleotides, account for the majority (98%) of the ncRNAs. Currently, about 30,000 different lncRNA transcripts are belived to exist in the human genome [2]. Since most lncRNAs are transcribed by RNA polymerase II (RNAP II), they share some similarities with mRNAs, such as poly-adenylation and the presence of 5′-cap structure. Just like mRNAs, lncRNAs form secondary structures, undergo post-transcriptional processing (i.e., 5’-cap structure, polyadenylation) and splicing [3], present in the nucleus, cytosol, and mitochondria [4], and can have tissue-specific expression patterns. 

lncRNAs have been shown to play a pivotal role in a wide range of cellular processes such as gene expression, translation regulation, splicing, chromosomal organization and X chromosome silencing [5,6,7]. Besides, specific lncRNAs are known to be dysregulated in various diseases, such as cancer, neurological diseases, and diabetes [8]. Considering their extensive roles in both health and disease, a better understanding of the functions of lncRNAs in the regulation of cellular events is needed. 

In this review, we aim to discuss the role of lncRNAs in the regulation of protein translation by controlling translational factors and signaling pathways. Furthermore, because translational regulation is often dysregulated in cancer cells, we also briefly summarize the role of lncRNAs in tumorigenesis and cancer progression as tumor promoters or tumor suppressors.

## 2. An Overview of the Characteristics of LncRNAs

Although lncRNAs were initially assumed as transcriptional noise or genomic “junk” [9,10], studies later revealed that they play vital roles in the regulation of various cellular processes, such as cell division, proliferation, differentiation, cell cycle, cell death, and metabolism [11,12,13,14,15]. Recent reports indicated some lncRNAs have a small open-reading frame (sORFs/smORFs) and are associated with ribosomes, suggesting their protein-coding potential [16,17,18,19,20]. In fact, recent studies showed that a small number of lncRNAs are capable of encoding small proteins called micropeptides (less than 100 amino acids) that are involved in the regulation of various biological processes [21].

Initial studies suggested that lncRNAs were thought of as unstable transcripts. However, later studies demonstrated that the majority of 800 lncRNAs have half-lives greater than 16 h and are highly stable, while only a minority of lncRNAs have half-lives less than 2 h [22]. 

lncRNAs have been traditionally categorized according to their specific locations on the genome into five major groups, including antisense, sense, bidirectional, intronic, and intergenic RNAs [23]. In a recent report, a more detailed classification has been proposed to describe the diversity of lncRNAs. This new classification includes seven different groups of lncRNAs: (a) mRNA-like intergenic transcripts (lincRNAs), (b) anti-sense transcripts of protein coding genes (natural anti-sense transcripts -NATs-), (c) processed transcripts, (d) enhancer RNAs (eRNAs), (e) promoter upstream transcripts (PROMPTs), (f) small nucleolar RNA (snoRNA)-ended lncRNAs (sno-lncRNAs), and (g) circular intronic RNAs (ciRNAs) [24].

## 3. Regulatory Functions of LncRNAs Depending on Their Subcellular Location

Since lncRNAs are capable of interacting with nucleic acids (DNA, RNA) and proteins, they are involved in the regulation of diverse molecular processes such as epigenetic and (post)-transcriptional modifications, translational regulation, splicing and scaffolding [6,7,25]. These diverse functions of lncRNAs are closely associated with their cellular location. lncRNAs are predominantly found in the nucleus and cytoplasm [1], while some lncRNA transcripts can be localized in exosomes. Recent findings revealed that large quantities of lncRNAs are exported to the cytoplasm to display their vital regulatory functions in cytoplasmic processes [19,26,27]. Subcellular localization of lncRNAs is a tightly regulated process controlled by various factors, such as sequence and structural motifs [28]. 

Based on their location in the cell, lncRNAs are involved in different molecular processes. The nuclear lncRNAs are closely associated with chromatin structures and regulate gene expression by influencing diverse mechanisms such as transcriptional and epigenetic regulation of specific genes and pre-mRNA processing [29]. In contrast, cytoplasmic lncRNAs dominantly control the stability and translation of mRNAs [27]. For instance, lncRNAs such as MALAT1 and NEAT1 are predominantly found in the nucleus; DANCR and OIP5-AS1 are found mainly in the cytoplasm; TUG1, CasC7 and HOTAIR have both nuclear and cytoplasmic distribution [30]. Since the subcellular location determines the function of lncRNAs, in this section, we aim to highlight the regulatory functions of lncRNAs depending on their subcellular locations.

### 3.1. Cytoplasmic LncRNAs

Cytoplasmic lncRNAs control a wide range of cellular processes by interacting with miRNAs, mRNAs and proteins. They can reciprocally interact with miRNAs and affect the functions of miRNAs in various ways. lncRNAs can function as competing endogenous RNAs (ceRNA) to bind miRNAs and block miRNA-mRNA interactions. For instance, BACE1 (beta-secretase-1) mRNA expression has been shown to be inhibited by miR-485-5p [31]. BACE1-antisense lncRNA and miR-485-5p compete for the same binding site in the ORF of the BACE1 mRNA and BACE1-antisense lncRNA prevents the mRNA-miRNA interaction [31]. In the second mechanism of lncRNA-miRNA interaction, lncRNAs can act as miRNA sponges or decoys and attract miRNAs, competitively sequestering miRNAs away from the target mRNAs [32]. For instance, lncRNA GAS5 (Growth arrest-specific 5), a tumor suppressor, functions as a sponge by sequestering and decreasing oncogenic effects of miR-21 and inhibits the proliferation of cancer cells and induces apoptotic cell death [33,34]. Similarly, lncRNA TRPM2-AS acts as a sponge or a competitive endogenous RNA for tumor-suppressor miR-612 and consequently modulates the derepression of IGF2BP1 and FOXM1 [35]. Silencing of TRPM2-AS inhibited aggressiveness of tumors in gastric cancer patients (proliferation, metastasis, radioresistance), while its overexpression promoted progression of gastric cancer [35].

lncRNAs in cytoplasm are also involved in the modulation of turnover and translation of some specific mRNAs [27]. lncRNAs can prevent the formation of mRNA-miRNA complexes as abovementioned, or they can bind to RNA-binding proteins (RBPs) [36,37]. For instance, lncRNA LAST stabilizes mRNA levels of Cyclin D1 (CCND1) oncogene. lncRNA LAST promotes the binding of CNBP-RBP (CCHC-type zinc finger nucleic acid binding protein) to CCND1, resulting in increased expression of CCND1 by stabilizing its mRNA [38]. Morover, lncRNAs modulate protein stability by influencing to enhance or hinder access to the ubiquitin-dependent proteasomal degradation machinery [27]. A study showed that lncRNA-p21 levels were transcriptionally activated by HIF-1α (Hypoxia-inducible factor-1α) under hypoxic conditions, then lncRNA-p21 binds to both HIF-1α and VHL (von Hippel-Lindau) proteins to protect HIF-1α from VHL-mediated ubiquitination [39]. Furthermore, lncRNAs can promote the proteasomal degradation. For instance, lnc-β-Catm recruits EZH2 to catalyze K49 methylation of β-catenin which inhibits phosphorylation and ubiquitination of β-catenin and promotes its stability [40].

### 3.2. Nuclear LncRNAs

Some of the lncRNAs are located in the nucleus to regulate gene expression by modulating chromatin organization, RNA processing and transcription [41,42,43,44,45]. The modulatory roles of lncRNAs on gene expression can be either cis- or trans-acting [41] and could negatively or positively affect the expression of target gene. 

## 4. Acting Mechanisms of LncRNAs in the Regulation of Translation 

### 4.1. Overview of Protein Translation Process

Protein translation is a highly complex process, comprising three steps (initiation, elongation, translation) and each step requires dynamic and efficient interactions between a great number of proteins, RNAs and ribosome.

The initiation process consists of two main steps. The first step involves the formation of the pre-initiation complex, and the second step is the assembling of this complex to the large subunit of the ribosome [46]. The initiation step begins with the formation of a ternary complex (eIF2-GTP-Met-tRNA), then the complex binds to small subunit (40S) of ribosome and assembles a pre-initiation complex by binding to other initiation factors (eIF1, eIF1A, eIF3, and eIF5) [47,48]. Before the pre-initiation complex directs to the 5′ end of mRNA, eIF4F complex, which is formed by eIF4E (cap-binding protein), eIF4G (scaffold protein) and eIF4A (helicase), bind to the 5′ end of mRNA to unwind and activate it [46,49]. The formation of eIF4F complex is maintained by some other initiation factors, eIF4B and eIF3. The pre-initiation complex then scans the mRNA until it recognizes a start codon [50]. Once the start codon is recognized, eIF5 and eIF5B promote hydrolysis of eIF2-bound GTP, releasing of eIFs from the complex and joining to the large subunit of the ribosome [51]. Following the initiation step of translation, met-tRNA reaches the P (peptidyl)-site of the 80S ribosome awaiting amino acids for elongation of the peptide chain.

The elongation step of translation requires the recruitment of aminoacyl-tRNA to the A (aminoacyl)-site of ribosome through GTP-bound eukaryotic elongation factor 1A (eEF1A). Although there is no base-pairing between tRNA anticodon and A-site codon, tRNA generates a codon-anticodon helix by remodeling itself [52] and stabilizes the ternary complex (aa-tRNA-eIF1A-GTP) [53]. Base-pairing interactions between A-site codon and aa-tRNA anticodon induce hydrolysis of GTP by eEF1A, which is then released from the A-site of the ribosome. eEF1A-GDP complex is recycled by eEF1B. Following the transfer of aa-tRNA to the A-site, a conformational change occurs in the ribosome which facilitates the formation of peptide bond between the aa-tRNA and the tRNA carrying the Met-tRNA at the P site. A GTPase (eEF2) binds to the A-site of the ribosome, hydrolyzes GTP and stimulates a conformational change in the ribosome resulting in movement of the ribosome one codon further. After the translocation of the ribosome, the A-site becomes empty and can accept the next aa-tRNAs to start a new cycle of elongation [52].

The last step of protein translation is termination, which begins when a stop codon (UAA, UGA, or UAG) reaches the A-site of the ribosome. Two types of release factors, eRF1 and eRF3, are involved in the termination process [54,55,56]. eRF1 is responsible for the recognition of stop codon and stimulation of peptide release, while eRF3 binds to eRF1 and triggers eRF1-mediated peptide release via GTPase activity [56,57]. The ternary complex (eRF1-eRF3-GTP) then binds to the ribosomal pre-termination complex and eRF3 hydrolyses GTP to release polypeptide [58].

### 4.2. Regulation of Translational Factors by LncRNAs

#### 4.2.1. Inhibitory Roles of LncRNAs in Translation through Regulation of Translation Factors

A growing body of evidence demonstrates that lncRNAs can regulate each step of translation by regulating the expression and the function of translation factors. For instance, lncRNA GAS5 is involved in the regulation of apoptosis and cell proliferation. A study performed with lymphoma cells showed that GAS5 interacts with the translation initiation complex, eIF4F, by directly binding to eIF4E and decreasing the translation of c-Myc [37]. Similarly, lncRNA RP1-5O6.5 has been shown to interact with eIF4E and prevents binding of eIF4E to eIF4G, leading to inhibition of translation of p27kip1, which negatively regulates Snail levels in breast cancer cells [59]. lncRNAs SNHG1 and SNGH4 are capable of binding to eIF4E and dysregulate the function of eIF4E in mantle cell lymphoma cells [60]. In the other example, lncRNA treRNA has been shown to interact with ribonucleoproteins (RNPs) (hnRNP K, FXR1, FXR2, PUF60, and SF3B3) and form treRNA-RNP complex which suppresses the translation efficiency of E-cadherin by binding eIF4G1 [61]. A brain-specific lncRNA, BC1, has been reported to interact with eIF4A and poly(A)-binding protein (PABP) and negatively regulate translation process [62,63]. lncRNA GAPLINC is overexpressed in non-small lung cancer cells and it increases eEF2K expression (a negative regulator of eEF2) by acting as a sponge for miR-661 [64]. In the other study, lncRNA FOXD1-AS1 was shown to bind to eIF5A, however it did not change the mRNA expression levels, suggesting that FOXD1-AS1 can involve in the post-translational regulation [65]. Overall, these studies suggested that lncRNAs can play an important inhibitory roles in mRNA translation through regulation of translation factors.

#### 4.2.2. LncRNAs Positively Regulate Protein Translation

Some lncRNAs have been reported to positively regulate protein translation. For instance, lncRNA SRA enhanced Wnt/β-catenin signaling pathway by increasing the expression of eIF4E-binding protein 1 (eIF4E-BP1) and contributed to the aggressive characteristics of endometrial cancer [66]. Another study showed that lncRNA MCM3AP-AS1 enhances the expression of eIF4E by acting as a sponge for miR15a, which supresses eIF4E expression and contributes to doxorubicin resistance in Burkitt lymphoma cells through MCM3AP-AS1/miR-15a/eIF4E axis [67]. Similarly, lncRNA SNHG12 enhanced the invasion of human vascular smooth muscle cells by serving as a sponge of miR-766-5p and influencing the miR-766-5p/eIF5A axis [68]. In the other study, a Y-linked lncRNA, LINC00278, was found to encode a micropeptide called YY1BM which led to a decrease in the expression of negative regulator of translation, eEF2K [69]. The functions of lncRNAs on translational factors are summarized in Table 1. 

### 4.3. LncRNAs Involved in Signaling Pathways Regulating Protein Translation

The PI3K/AKT/mTOR is one of the major signaling pathways known to regulate vital cellular processes including cell proliferation, growth, survival, metabolism and protein translation. The role of PI3K/AKT/mTOR and MAPK pathways in the regulation of translational machinery are well documented and they are frequently overactivated in most types of cancer [70]. Both pathways involve the mechanistic target of rapamycin (mTOR) to regulate a variety of components of the translational machinery in homeostasis, their dysregulation results in aberrant translation which is often detected in diabetes, neurological disorders, and cancer [71,72,73,74]. The MAPK family consists of a serine/threonine kinases, that includes ERKs, JNKs and p38/SAPKs [75]. Especially the MAPK/ERK signaling pathway is amongst the most well-studied, signaling and dysregulating one-third of all human cancers [76]. 

PI3K/AKT/mTOR pathway regulates cell growth and proliferation by phosphorylating two downstream targets which are 4E-BP1 and ribosomal protein S6 kinase (S6Ks). mTOR complex I (mTORC1) controls translational activation by phosphorylating eIF4E inhibitor, 4E-BP1, which releases eIF4E to interact with initiation complex (eIF4F) [77]. S6K protein requires sequential phosphorylations at multiple serine/threonine sites and mTORC1 regulates its activation by phosphorylation. Once S6K is activated, it phosphorylates and activates eIF4B, which increases the recruitment of eIF4B to eEF4A and enhances translation [78]. Besides, S6K and mTORC1 signaling pathways can phosphorylate EF2-Kinase (EF2K) and decrease its sensitivity to Ca/Calmoduline for its activation [79]. Similarly, eEF2K activity is negatively regulated by MAPKs and their downstream effectors, reducing phosphorylation of eEF2, leading to increased translation by promoting peptide elongation phase of protein systhesis [80,81]. Considering the significant regulatory roles of PI3K/AKT/mTOR and MAPK signaling pathways in protein translation, regulation of their activity by lncRNAs indicate that the lncRNAs are involved in controlling protein translation through regulation of these key signaling pathways. For instance, lncRNA UASR1 promotes cell growth and migration of breast cancer cells by regulating AKT/mTOR pathway [82]. In these cells, active mediators of this pathway such as p-AKT, p-TSC2, p-4EBP1 and p-p70S6K are increased by overexpression of UASR1. Thus, UASR1 plays an oncogenic role in breast cancer cells through activation of the AKT/mTOR signaling pathway. Another lncRNA H19 is overexpressed in colorectal cancer tissues and it promotes the activity of PI3K/AKT pathway by acting as a ceRNA and regulating some components of this pathway. H19 regulates various cancer-related mRNAs (such as (AKT3, CSF1, MET, COL1A1) by competitively sponging various miRNAs. Knockdown of H19 reduced protein level of MET, ZEB1, and COL1A1 in vitro [83]. The other study showed that H19 inhibits mTORC1-mediated 4E-BP1 phosphorylation, but it does not affect the activation of S6K1 [84]. lncRNA CASC9 has been shown to suppress apoptosis and promote aggressiveness of oral squamous cell carcinoma cells by activating the AKT/mTOR pathway [85]. 

In contrast, some lncRNAs might negatively regulate the abovementioned pathways. For instance, lncRNA FER1L4 suppresses cell proliferation and metastasis through downregulating the expressions of PI3K and AKT in lung cancer cells [86]. Overall, lncRNAs can regulate signaling pathways involved in translational control that is an integral part of these survival adaptive pathways in normal and cancer cells. Some of these regulatory lncRNAs and their functions on signaling pathways are summarized in Table 2. 

### 4.4. LncRNAs in Cancer

#### 4.4.1. LncRNAs Can Contribute Hallmarks of Cancer 

**Figure 1:**
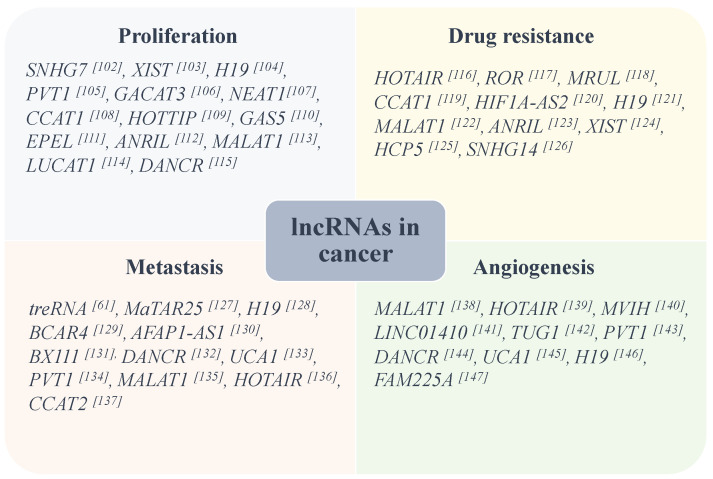
Some lncRNAs are identified to be involved in aggressive characteristics of some common types of cancers [61,102,103,104,105,106,107,108,109,110,111,112,113,114,115,116,117,118,119,120,121,122,123,124,125,126,127,128,129,130,131,132,133,134,135,136,137,138,139,140,141,142,143,144,145,146,147].

Deregulation of mRNA translation is commonly observed in malignant cells and is considered as a critical factor contributing to cancer initiation, tumorigenesis, and progression. Because lncRNAs play critical roles in the regulation of a wide range of cellular processes, their dysregulation is associated with cell proliferation, survival, tumorigenesis and progression of various cancers, and aberrant expression of lncRNAs can contribute to the hallmarks of cancer. Reprograming of the translation machinery in cancer cells is important function of the key oncogenic signalings, promoting cellular transformation. Increased activity of translational machinery has been shown to be critical in many cancer cells, including breast [97], pancreatic [98], liver [99], and colorectal cancer [100], and leukemia [101]. Thus, lncRNA-mediated regulation of protein translation plays an important role in promoting oncogenic signaling, and specific targeting of these lncRNAs holds promise for developing highly targeted therapies in cancer and other human diseases. Figure 1 illustrates some of the lncRNAs that are involved in tumorigenesis and cancer progression.

#### 4.4.2. The Functions of LncRNAs in Regulating Translation of Cancer-Related Proteins

As mentioned above, various lncRNAs are involved in the regulation of hallmarks of cancer, suggesting that they have potential regulatory roles in cancer-related protein tranlation. Since we have already summarized the roles of some lncRNAs on PI3K/AKT/mTOR and MAPK pathways in Table 2, here we briefly focus on the interaction between lncRNAs and translation, promoting the aggressive tumor characteristics.

An example of a lncRNA that is well-known to be associated with cancer is MALAT1. MALAT1 was shown to upregulate the expression of glycolytic genes which contributes the aggressive characteristics of hepatocellular carcinoma cells. MALAT1 regulated the glucose metabolism of hepatocellular carcinoma cells by enhancing translation of metabolic transcription factor TCF7L2 through mTORC1–4EBP1 axis [87]. lncRNA NEAT1 represents another example of lncRNAs that contribute to the aggressiveness of non-small cell lung cancer by enhancing eIF4G2 via miR-582-5p sponging effects [148]. Similarly, lncRNA RP11-284P20.2 enhanced c-met mRNA translation by recruiting eIF3b to c-met and thus promoted proliferation and invasion of hepatocellular carcinoma cells [149]. In prostate cancer, lncRNA UCA1 levels were found to be positively correlated with eIF4G1 levels. UCA1 enhances eIF4G1 levels via sponging miR-331-3p, while knockingdown of UCA1 sensitizes prostate cancer cells to radiotherapy by suppressing eIF4G1 expression via miR-331-3p/eIF4G1 axis [150]. In another study, lncRNA GAPLINC increased the eEF2K expression by serving as a sponge for miR-661, thereby promoted proliferation and progression of non-small cell lung cancer [64]. 

lncRNAs can also regulate translation process by interacting with the ribosome or ribosome-related proteins. For example, lncRNA ZFAS1 was shown to interact with a small 40S subunit of the ribosome in breast cancer cells. The study showed that ZFAS1 did not regulate translation process directly. Instead, the lncRNA was increased during the ribosome biogenesis indicating its role in regulating the ribosome production and assembly [151]. In neuroblastoma cells, it was shown that lncNB1 enhanced E2F1 protein synthesis and N-Myc stability by binding the ribosomal protein RPL35 [152]. 

**Figure 2:**
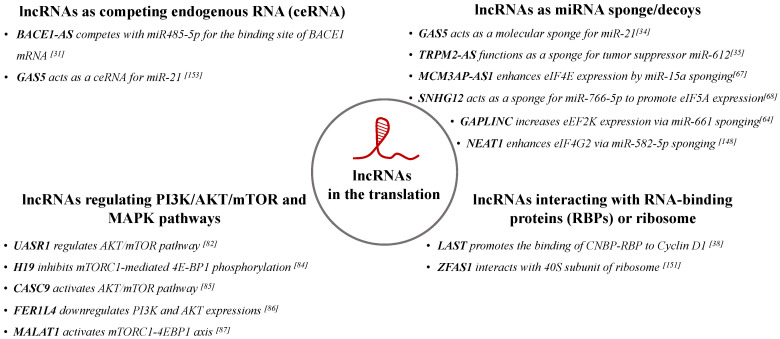
The mechanisms of action of lncRNAs on the regulation of cancer-related protein translation [31,34,35,38,64,67,68,82,84,85,86,87,148,151,153].

Overall, an emerging body of evidence suggests that lncRNAs play important roles in the regulation of protein translation process. They can enhance or suppress translation via several mechanisms, including through interacting with the ribosome-associated proteins, sponging miRNAs, and competing with endogenous RNAs. Their mechanisms of action and some examples are summarized in Figure 2. 

## 5. Conclusions

Advances in high throughput technologies resulted in the identification of a large number of lncRNAs. Although thousands of lncRNAs have been identified in the genomes of higher eukaryotes, our understanding of the mechanisms by which lncRNAs exert their precise function for most of them remains unknown. Elucidating the function of these lncRNAs is expected to provide deeper insight into the molecular mechanisms regarding their function in human diseases, including cancer and the interaction of lncRNAs with other molecules may help to design novel strategies. Accumulating evidence indicates that lncRNAs display pivotal roles in the regulation of almost every cellular process by binding to the target proteins, mRNAs, miRNA, and/or DNAs, indicating the complicated roles of lncRNAs. Recent findings revealed that lncRNAs can play important roles in the pathogenesis of human cancers, contributing to tumor growth and progression. Thefore, a better understanding of the role of lncRNAs is needed to elucidate the missing links in the molecular mechanims involved in human diseases, including cancer. 

## Figures and Tables

**Table 1:** The list of long non-coding RNAs (lncRNAs) involved in regulation of translational factors [37,59,60,61,62,63,64,65,66,67,68,69].

LncRNA	Translation Factor	Function	Reference
GAS5	Binds to eIF4E and prevents formation of initiation complex (eIF4F)	Decreases translation of c-Myc	[37]
RP1-5O6.5	Interacts with eIF4E and prevents binding to eIF4G	Promotes breast cancer metastasis by inhibiting translation of p27Kip1	[59]
SNHG1 and SNGH4	Bind to eIF4E and dysregulate it	Enhance translation and contribute aggressiveness of lymphoma cells	[60]
treRNA	Promotes the formation of a treRNA-associated protein (treRNP) complex and suppresses translation by binding to eEIF4G1	treRNP complex reduces translation efficiency of E-cadherin and decreases tumor metastasis	[61]
BC1	Interacts with eIF4A and poly(A)-binding protein (PABP)	Represses translation	[62,63]
GAPLINC	Positively regulates eEF2K expression by sponging miR-661	Promotes tumorigenesis of non-small cell lung cancer cells	[64]
SRA	Binds and increases the expression of eIF4E-binding protein 1 (eIF4E-BP1)	Increases the activity of Wnt/ β-catenin signaling and promotes aggressive characteristics of endometrial cancer	[66]
MCM3AP-AS1	Positively regulates the expression of eIF4E by using miR15a as a sponge	Promotes translation and contributes doxorubicin resistance	[67]
SNGH12	Binds to miR-766-5p, which is a negative regulator of eIF5A	Targets miR-766-5p/eIF5A axis and enhances invasion of vascular smooth muscle cells	[68]
LNC00278	Decreases eEF2K expression	Micropeptide of lncRNA, YY1BM, represses the eEF2K/eEF2 axis	[69]

**Table 2:** lncRNAs in the regulation of signaling pathways and their roles in various cancers [87,88,89,90,91,92,93,94,95,96].

LncRNA	Target	Function	Reference
MALAT1	mTOR signaling	Improves glucose metabolism to contribute aggressiveness in hepatocellular carcinoma cells	[87]
HOXB-AS3	PI3K/AKT signaling	Increases proliferation, migration, and invasion of lung cancer cells	[88]
AK023391	PI3K/AKT signaling	Promotes tumorigenesis and invasion of gastric cancer	[89]
LOC101928316	PI3K/AKT/mTOR signaling	Inhibits cell proliferation, invasion and tumorigenesis of gastric cancer cells	[90]
UCA1	PI3K/AKT signaling	Promotes cell proliferation and inhibits apoptosis in retinoblastoma cells	[91]
OECC	PI3K/AKT/mTOR signaling	Increases proliferation, migration and invasion of lung cancer cells	[92]
GAS5	PTEN/PI3K/AKT signaling	Suppresses proliferation and invasion of osteosarcoma cells and promotes PTEN expression by sponging miR-23a-3p	[93]
LINC01503	MAPK/ERK signaling	Increases proliferation and tumor forming-ability of hepatocellular carcinoma cells	[94]
ST8SIA6-AS1	p38 MAPK signaling	Promotes proliferation, migration and invasion of breast cancer cells	[95]
FENDRR	p38 MAPK signaling	Inhibits cell proliferation and induces apoptosis in hepatocellular carcinoma cells	[96]
